# Targeting Redox Regulation as a Therapeutic Opportunity against Acute Leukemia: Pro-Oxidant Strategy or Antioxidant Approach?

**DOI:** 10.3390/antiox11091696

**Published:** 2022-08-29

**Authors:** Alessandro Allegra, Alessandro Tonacci, Laura Giordano, Caterina Musolino, Sebastiano Gangemi

**Affiliations:** 1 Division of Hematology, Department of Human Pathology in Adulthood and Childhood “Gaetano Barresi”, University of Messina, 98125 Messina, Italy; 2 Clinical Physiology Institute, National Research Council of Italy (IFC-CNR), 56124 Pisa, Italy; 3 Allergy and Clinical Immunology Unit, Department of Clinical and Experimental Medicine, University of Messina, 98125 Messina, Italy

**Keywords:** oxidative stress, acute myeloid leukemia, acute lymphoblastic leukemia, reactive oxygen species, mitochondria

## Abstract

Redox adaptation is essential for human health, as the physiological quantities of non-radical reactive oxygen species operate as the main second messengers to regulate normal redox reactions by controlling several sensors. An abnormal increase reactive oxygen species, called oxidative stress, induces biological injury. For this reason, variations in oxidative stress continue to receive consideration as a possible approach to treat leukemic diseases. However, the intricacy of redox reactions and their effects might be a relevant obstacle; consequently, and alongside approaches aimed at increasing oxidative stress in neoplastic cells, antioxidant strategies have also been suggested for the same purpose. The present review focuses on the molecular processes of anomalous oxidative stress in acute myeloid and acute lymphoblastic leukemias as well as on the oxidative stress-determined pathways implicated in leukemogenic development. Furthermore, we review the effect of chemotherapies on oxidative stress and the possibility that their pharmacological effects might be increased by modifying the intracellular redox equilibrium through a pro-oxidant approach or an antioxidant strategy. Finally, we evaluated the prospect of varying oxidative stress as an efficacious modality to destroy chemoresistant cells using new methodologies. Altering redox conditions may be advantageous for inhibiting genomic variability and the eradication of leukemic clones will promote the treatment of leukemic disease.

## 1. Introduction

### General Consideration on Oxidative Stress

Reactive oxygen species (ROS) are a type of functional molecule that contains oxygen and is biologically generated during cell metabolism. As such, they are implicated in controlling physiological chemical reactions, cell activities, and cell adaption [1,2,3].

ROS can be of exogenic or endogenic origin as cellular events produce ROS via cytochrome p450 activity, mitochondria-derived transport effects, and inflammatory events involving different type of cells such as neutrophils, macrophages, and eosinophils [4].

Several systems can cause increased ROS generation, such as cytochrome P450 monooxygenase, xanthine oxidoreductase, cyclo-oxygenase, uncoupled nitric oxide synthase, and NADPH oxidase. Amongst intracellular structures, mitochondria are the main source of ROS production owing to their activity in oxidative phosphorylation and aerobic metabolism. The formation of mitochondrial ROS occurs at complex I and complex III of the electron transport chain because of the escape of electrons that fail to reach complex IV and generate the superoxide radical anion. However, this anion may also be produced during fatty acid oxidation or through the action of xanthine oxidase. A different source of ROS in mitochondria is correlated with the cytochrome catalytic cycle through which a huge amount of substances, such as xenobiotics and hormones, are processed to generate the superoxide radical and H_2_O_2_ as by-products. Peroxisomes constitute a further source of ROS as several metabolic processes in these organelles generate a wide range of ROS via the actiom of xanthine oxidase and xanthine dehydrogenase. The latter is implicated in purine catabolism and in the generation of reactive nitrogen species. Finally, the diverse structure involved in ROS generation is the endoplasmic reticulum (ER), which has the additional role of producing, folding, and assembling proteins. Oxidative protein folding is regulated by different ER oxidoreductases, including ERp72, ERp57, and protein disulfide isomerase, which are oxidized by endoplasmic reticulum oxidoreductin-1 to produce H_2_O_2_ [5,6,7].

In hematopoietic stem cells (HSCs), ROS are produced by the mitochondria via the effect of reduced nicotinamide adenine dinucleotide phosphate (NADPH) oxidases (NOX) or through the metabolism of different compounds such as xanthine oxidase or polyamine [8].

The cellular effects of ROS depend on their quantity; in low amounts, ROS are useful to stimulate cell growth, while increased levels induce cell injury or death. Usually, an equilibrium between the production and removal of ROS is provided by antioxidants such as glutathione (GSH) and thioredoxin.

The thioredoxin complex is an essential element of the antioxidant system, which eliminates ROS to preserve the intracellular redox equilibrium. It is composed of thioredoxin, thioredoxin reductase, and NADPH [9,10,11,12,13,14], and any of these elements have been reported to be altered in several tumors, including leukemia. Oxidative stress (OS) occurs when the antioxidant system is incapable of neutralizing pro-oxidants, provoking injury to different cell elements and stimulating particular pathways [15]. The more relevant phenomena by which OS induces cellular damage are the oxidative alteration of proteins and the lipid peroxidation of cellular membranes [16]. It was proved that lipid peroxidation induces alterations in nuclear and mitochondrial DNA (mtDNA) and genomic changes, whereas the duplication of injured DNA before repair causes genomic instability [17].

An increased probability of tumor in different tissues has been correlated to redox alterations and several experimentations have discovered the process by which long-lasting oxidative stress can induce protracted inflammation, which can cause several chronic pathologies including tumors. Moreover, a huge number of transcription factors may be expressed after the onset of oxidative stress, stimulating the activation of more than five hundred different genes, including those able to regulate the production of cell cycle controlling factors, chemokines, cytokines, and growth factors [18].

Previous studies performed at our and other laboratories have demonstrated the existence of a relationship between oxidative stress and hematological malignancies such as multiple myeloma, chronic lymphocytic leukemia, Hodgkin’s lymphoma, and chronic myeloproliferative neoplasms [19,20,21,22,23,24,25,26,27,28].

The present review focuses on the molecular processes provoking abnormal ROS production in acute myeloid and lymphoblastic leukemia as well as on the redox- supported pathways implicated in the onset of leukemias. Furthermore, we evaluated the effects of chemotherapy on oxidative stress and the possibility that the pharmacological effects may be at least partially caused by modifying the cellular redox status of leukemic cells. Finally, we discuss the possibility of modifying the oxidative equilibrium through a pro-oxidant or an antioxidant strategy as an effective way to eradicate chemo-resistant cells and the role of new methodologies, such as nanotechnologies and the use of cold atmospheric plasma, in treating leukemic disease.

## 2. Oxidative Stress and Acute Myeloid Leukemia

Acute myeloid leukemia (AML) is the most commonly diagnosed acute leukemia, and in older subjects, AML leads to a bad prognosis. The etiology of AML implicates the occurrence of the malignant transformation of hematopoietic stem cells that, after different genomic mutations, finally causes the full-blown disease. Although the pathogenesis of AML remains undetermined, exposure to genotoxic factors could participate to the onset of this disease. From a disease biology viewpoint, AML is an extremely heterogeneous condition comprising biological and clinical features with several genetic, epigenetic, and phenotypic differences, not only between the various forms of leukemia, but also between one patient and another. Furthermore, different clonal cells co-occur in the majority of AML subjects. Single subclones are typified by specific genetic and epigenetic alterations, which participate in the establishment of different biological behaviors. As a consequence, despite huge progress in our comprehension of the biology of AML, the results of treatment still appear largely unsatisfactory. The identification of factors capable of modifying cell proliferation and death, or the response to chemotherapeutics, could help the management of patients with AML. However, due to the intrinsic heterogeneity of the disease, particular caution must be employed in the analysis of the results, since these are strictly dependent on the cell type used and the experimental conditions adopted.

The analysis of the oxidative stress alterations present in these cells could however open new scenarios able to favor our understanding of the biological phenomena of AML and support the identification of new possible interventions [29,30].

Metabolic modification is recognized as a classical characteristic of tumors, and in this situation, the abnormal metabolic condition induces an increased oxidative stress in tumor cells [31].

**Figure 1:**
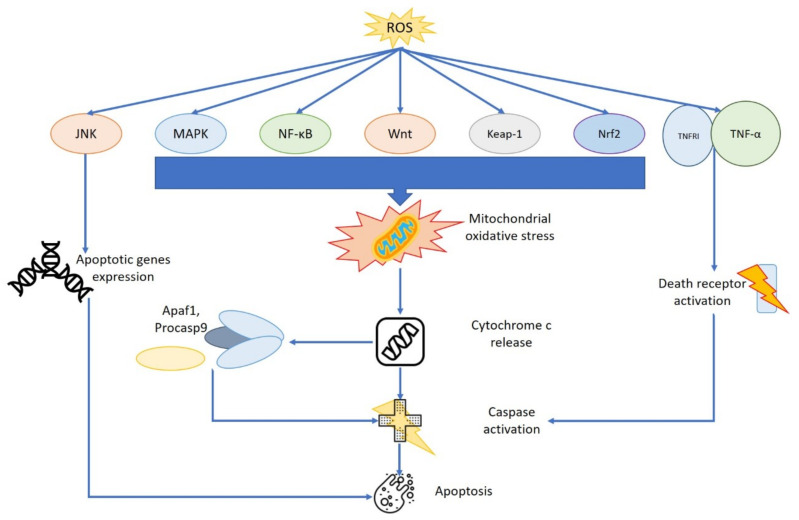
Mechanisms of increased apoptosis as consequence of oxidative stress. Increased concentrations of ROS stimulate several different pathways able to modify mitochondrial oxidative stress, activate the caspase system, and induce programmed cell death. NF-kB nuclear factor kappa-B; Wnt Wingless and Int-1; Nrf2 nuclear factor (erythroid-derived 2)-like 2; MAPK mitogen-activated protein kinase; Keap1 Kelch-like ECH-associated protein 1; Apaf1 apoptosis protease-activating factor 1.

Similar to normal cells, the consequences of ROS also differ with respect to their amount in clonal cells. Recently, it was reported that a large quantity of ROS inhibits neoplastic proliferation and stimulate programmed cell death, which is the main effect of most chemotherapeutic drugs [32]. Furthermore, increased concentrations of ROS stimulate several different pathways, such as nuclear factor kappa-B (NF-κB), Wingless and Int-1 (Wnt), nuclear factor (erythroid-derived 2)-like 2 (Nrf2), mitogen-activated protein kinase (MAPK), and Kelch-like ECH-associated protein 1 (Keap1), which are essential factors in the stimulation of apoptosis [33]. This effect may be due to an increase in mitochondrial oxidative stress, which in turn induce the release of cytochrome c into the cytosol, determining a stimulation of caspases (Figure 1).

A different mechanism for the induction of apoptosis involves gathering of misfolded proteins resulting from increased oxidative stress, which can modify the activity of the endoplasmic reticulum. Neoplastic cells respond to ER stress via the stimulation of the unfolded protein response, which is made of several protective signaling pathways through which stressed cells try to interrupt protein translation, easing ER-associated protein degradation. However, if stressed cells are unsuccessful, the unfolded protein response will stimulate programmed cell death, which is due to the triggering of ER-resident caspases, mainly, caspase 4. In fact, in an in vitro study to discover a caspase that was distinctively implicated in ER stress, authors assessed human colon cDNA libraries employing the mouse caspase-12 gene as a probe. Caspase-4 was replicated, and the researchers evaluated the subcellular positioning of endogenous caspase-4 in SK-N-SH neuroblastoma cells. Using immunofluorescence microscopy, the detection of the immunostaining profile of caspase-4 exactly coincided with that of ER indicators such as GRP94 and GRP78. These findings indicate that caspase-4 was placed essentially to the ER, and to the mitochondria. Furthermore, they evaluated the cleavage of pro-caspase-4 after different apoptotic factors, and they evidenced that the cleavage of pro-caspase-4 was induced by the administration of tunicamycin and thapsigargin, both of which are able to induce ER stress. In contrast, when cells were treated with non-ER stress factors such as etoposide or staurosporine, cleavage elements of pro-caspase-4 were not detected [34].

In contrast, at low amounts, ROS reduce programmed cell death and stimulate cell proliferation, motility, and the onset of chemoresistance. Oxidative DNA injury may cause different phenomena, such as alteration in transcription, replication mistakes and genomic variability, all of which are correlated with leukemogenesis. Mitochondrial DNA appears to be more sensitive to oxidative stress-induced alterations as it does not possess DNA repair enzymes. Furthermore, oxidative stress induces autophagy in the tumoral marrow milieu, inducing the stromal production of recycled nutrients. These factors aid mitochondrial biogenesis in clonal cells, augmenting their proliferation through an energy imbalance. Finally, anoikis is a condition by which the programmed cell death of normal cells is stimulated after the loss of cell matrix attachment. ROS have been reported to cause anoikis resistance, thereby easing tumor diffusion [18].

This concept applies not only to solid neoplasms, but also to hematological neoplasms. In leukemia, an increase in ROS and antioxidants, as a defense response, suggest that the presence of OS is able to support leukemogenesis [35].

Reports have demonstrated that more than 60% of AML clonal cells generate enormous amounts of NOX-originated ROS, stimulating cell growth and the persistence of AML cells [36]. The increased ROS concentrations occur concomitantly with decreased concentrations of GSH and peroxiredoxin [37], showing that NOX-originated ROS induce effects capable of inhibiting signaling that would generally reduce this response [38].

**Figure 2:**
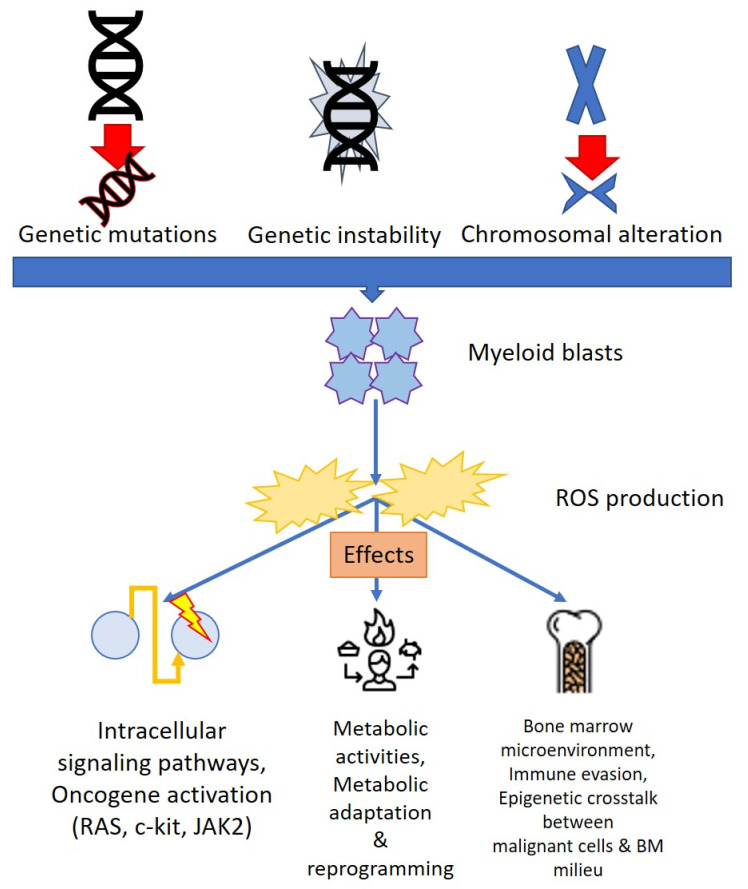
Effects of ROS on leukemogenesis. Chromosomal, genetic, and epigenetic alterations of the leukemic cells co-occur to determine the abnormal production of ROS capable of inducing alteration of the intracellular signaling pathways, modification of the intracellular metabolic activities (energy balance and chemoresistance), and an effect on the cells of the medullary microenvironment (angiogenesis, bone homeostasis, extracellular matrix remodeling, immune activity).

Furthermore, ROS generated by leukemic blasts are implicated in the control of the bone marrow (BM) microenvironment, thus supporting leukemia progression [39]. Indeed, in the leukemic BM milieu, NADPH oxidase-2 (NOX2)-derived ROS stimulate mitochondrial transport between leukemic blasts and BM stromal cells via tunneling nanotubules, increasing metabolic alterations in AML cells [40] (Figure 2).

Of particular interest is the result that the generation of ROS by leukemic cells appears quite different according to their type; this can play a role in the prognostic stratification of these patients. Fms-like receptor tyrosine kinase 3-internal tandem duplication (FLT3-ITD), c-Kit, and Ras are essential leukemic oncogenes produced by genomic alterations. FLT3-ITD is the most frequent genetic mutation found in AML and is present in 30% of AML subjects [41]. This mutation induces ROS increase via (NOX) and fosters the growth of AML cells, inducing survival signals [42]. Similarly, Ras alteration, present in 20% of AML patients, induces cell alterations via NOX-driven ROS, thus stimulating CD34+ cell proliferation, which is growth factor-independent [43]. Thus, the interpretation of the results may vary between the different studies, and alongside the direct action of ROS on cell proliferation, other mechanisms may intervene to justify the phenomenon. Using oxidase inhibitors and antioxidants, authors found that augmented ROS generation by these cells did not participate in their greater survival; rather, ROS supported their factor-independent proliferation. Although Ras-induced ROS generation specifically stimulated the p38MAPK oxidative stress response, this failed to cause the production of the cell cycle inhibitor, p16INK4A; instead, ROS stimulated the expression of D cyclins [43].

Finally, c-Kit also has an effect on ROS generation by controlling NOX functions [44]. However, other factors may be important, such as B-cell lymphoma 2 (Bcl-2), which functions as an oncogene in AML. Although any experimentations reported an inhibitory effect performed by the molecule on oxidative stress via the stimulation of antioxidant molecules in tumor cells [45], other studies have demonstrated that Bcl-2 can stimulate ROS generation in leukemia cells. Bcl-2-induced ROS generation in leukemia cells is associated with increases in cellular oxygen utilization, cytochrome c oxidase/complex-IV activity, and mitochondrial respiration [46]. The discrepancies between the numerous studies could be due to the different experimental conditions.

In this sense, an alteration of the oxidative balance can enhance the risk stratification of patients with AML, and it is not impossible that data relating to oxidative stress will soon be included in new prognostic scores.

In fact, several systems able to control oxidative stress seem to be altered in AML subjects. For instance, the proteins S100A8 and S100A9, which consist of 93 and 114 amino acids, respectively, are frequently altered in the plasma and BM of AML patients. These alterations modify the outcome of leukemia patients, affecting the survival of leukemic cells. In particular, S100A8 and S100A9 can regulate the proliferation and the response to chemotherapy of leukemic cells via their effects on the control of OS, the regulation of intracellular calcium, the stimulation of programmed cell death, and the ability to influence autophagy and pyroptosis. Furthermore, S100A8/9 seems able to function through toll-like receptor four and receptor for advanced glycation end-products in hematopoietic stem cells by producing specific cytokines and modifying the immune response [47].

S100A8 and S100A9 can induce cell death via different systems, such as the stimulation of caspase 3-dependant apoptosis through a reduction in Zn^2+^, and the decrease in mitochondrial membrane potential, provoking the delivery of Smac/Diablo and Omi/HtrA2 that upset the equilibrium between pro- and anti-apoptotic signals [48,49]. In addition, as reported above, S100A8 and S100A9 have demonstrated to stimulate cell death by autophagy. The administration of S100A8 and S100AA9 induces the transfer of BNIP3 (a component of the Bcl2 group) to mitochondria and stimulates an increase in ROS, which induces mitochondrial injury and lysosomal stimulation, causing cell death through autophagy [50].

S100A8 and S100A9 can also increase pyroptosis by joining CD33 and TLR4 and increasing the generation of inflammasome elements, leading to the production of cytokines such as IL-18 and IL-1 beta. Actually, S100A9 can elicit pyroptosis in myelodysplastic BM mononuclear cells. In vitro, myelodysplastic hematopoietic progenitor cells present the augmented expression of inflammasome proteins and display activated NLRP3 elements that induce the stimulation of caspase-1, the production cytokines such as IL-1β and IL-18, and pyroptotic cell death. As for the mechanisms involved, pyroptosis is elicited by the alarmin S100A9 that is reported to be augmented in myelodysplastic hematopoietic stem cells. Furthermore, S100A9-mediated signaling stimulated NOX, augmenting ROS concentrations that induced cation inflow, cell bulging, and β-catenin stimulation. Remarkably, the silencing of NLRP3 or caspase-1, the deactivation of S100A9, or pharmacologic blockade of NOX or NLRP3 inhibited ROS production, pyroptosis, and were able to re-establish efficient hematopoiesis [51].

More complex and less clear are the effects exerted by metallothioneins (MTs), which are different intracellular molecules able to change the oxidative status of leukemic cells. MTS are a group of proteins with essential effects in detoxification, cell differentiation, programmed cell death, angiogenesis, and chemoresistance, as well as leukemogenesis [52,53,54,55].

Xin et al. showed that the production of metallothionein1X (MT1X) was increased in AML subjects, and that this amount had a prognostic value [56]. In vitro studies demonstrated that inhibiting MT1X reduced the growth of AML cells, restored sensitivity to drugs such as doxorubicin, and increased programmed cell death. Probably, these effects were due to the reduction in nuclear factor-κB (NF-κB) caused by the decrease of p65, and p-IκB-α. However, the study demonstrated that miR-376a-3p exerts a negative control on MT1X, and that miR-376a-3p increase reduced AML cell growth and fostered programmed cell death. In this situation, it is probable that the global results of the system overcome over the actions exerted on the redox equilibrium [56]. Therefore, it appears evident that the evaluation of a single operator on the oxidative stress may not be sufficient to clarify the final effects produced on neoplastic proliferation.

### 2.1. Modulation of Oxidative Stress to Treat AML Leukemia

Two completely different therapeutic strategies have been used in the adjustment of oxidative stress to attain the eradication of leukemic cells: the pro-oxidant approach and the antioxidant method. The first strategy has been favored in recent years according to the supposition that increasing ROS concentrations using chemotherapy or different ROS-generating factors fosters programmed cell death and leads to the amelioration of neoplastic disease.

In fact, chemotherapy alters the metabolic systems of leukemic cells, causing signal transmission anomalies, thus stimulating enhanced ROS generation.

The best-known therapeutic scheme for the treatment of acute myeloid leukemia is the so-called 3 + 7 protocol, containing anthracycline and cytarabine. The latter causes ROS increase in leukemia and normal cells and is also able to change the antioxidant system. These alterations cause OS and ultimately induce the onset of programmed cell death [57]. Analogously, daunorubicin, idarubicin, and mitoxantrone (anthracycline derivatives) can stimulate ROS generation by interrelating with molecular oxygen and causing direct DNA injury [58]. Moreover, these compounds may bind to free iron in cells, causing a Fenton reaction provoking a further increase in ROS.

In a diverse set of leukemia patients, such as patients affected by acute promyelocytic leukemia (APL), arsenic trioxide was also reported to increase ROS generation via NOX stimulation and thioredoxin reduction [59], and a recent in vitro experiment proved that leukemic cell death was correlated with enhanced OS and ROS generation. Noguera et al. investigated the toxic consequences of high-dose ascorbate, alone or combined with arsenic trioxide (ATO) in AML and APL cell lines and in primary blasts from AML and APL subjects. Several findings indicate that ascorbate functions as an antioxidant at low small levels but has pro-oxidant functions at higher levels. Cells were treated with different amounts of ascorbate, or ascorbate and ATO. The high-dose ascorbate/ATO treatment destroyed myeloid blasts, while saving CD34+ progenitors isolated from normal cord blood. As for the mechanism of cell destruction, it seemed to be correlated with augmented OS and increased ROS generation, which caused an increase in programmed cell death. These results were regressed by employing antioxidants. Furthermore, in the APL NB4 experimental model, high-dose ascorbate induced degradation of the PML and PML/RARA proteins through caspase stimulation, while the histone chaperone DAXX was engaged in re-constituted PML nuclear bodies [60].

However, we must bear in mind that long-lasting OS conditions due to chemotherapy may induce chemoresistance and cause further genetic instability in clonal cancer cells [61,62].

The modulation of OS could also be fundamental in reducing relapse after therapy and achieving minimal residual disease via an effect on leukemic stem cells (LSCs). In fact, even though, with the use of new drugs, the overall survival of AML patients has increased [63], during treatment LSCs may be located within the BM niche in a dormant condition, escaping the destructive effect of the chemotherapeutic drugs. In this sense, the protective effect of the BM niche on the remaining LSCs is the main reason for chemoresistance in AML [64]. Numerous experimental studies have demonstrated that pursuing the LSCs placed in the BM niche and altering the oxidative balance may be a novel approach to reduce leukemia relapse [65].

Nevertheless, normal hemopoietic stem cells (HSCs) are also extremely susceptible to an increased ROS concentration, and how to decrease the toxic consequences of HSCs while destroying LSCs constitutes the most relevant problem in pro-oxidant treatment of AML.

The possibility of exerting different effects on the two diverse types of cells is correlated to the differing metabolic status of normal stem cells and LSCs. As neoplastic cell growth in the BM niche results in anemia, a condition of severe hypoxia is produced within the BM niche, but leukemic cells are less sensitive to hypoxia with respect to normal HSCs. Goto et al. demonstrated that leukemic cells have better survival in a hypoxic milieu compared to HSCs by decreasing ROS production and increasing ROS elimination [66]. For these reasons, ROS concentrations are lower in LSCs, which is essential for their survival and the preservation of their stemness, while the ability to enhance ROS levels may stimulate the death of LSCs [67]. Recent experiments have confirmed that the self-renewal capability of LSCs is strongly correlated with the ROS concentration and OS in the cells. Herault et al. demonstrated that LSCs presented an increase of glutathione peroxidase by three-fold, and reduced ROS concentration, thus preserving the characteristics of stem cells [68]. Furthermore, when comparing the ROS concentrations of LSCs with the ROS levels of more primitive leukemic cells in a dormant condition, it was clear that the ROS level is greater in leukemic cells that are multiplying more rapidly [69].

These findings allow us to hypothesize that the increase in OS and the killing of AML cells, especially of LSCs, can also be obtained with substances other than conventional chemotherapy.

The mitochondria are the main players in intracellular ROS production and altering the mitochondria may be a convenient approach to modify the oxidative equilibrium of the leukemic cells, cause OS, and stimulate the programmed cell death of AML cells. Several mitochondrial regulators that can modify ROS production are evaluated for their possible effects in AML therapy. For instance, metformin, an antidiabetic medicine, has been reported to be able to decrease mitochondrial ATP production and increase ROS concentration [70]. Analogously, adaphostin, an adamantly derived tyrphostin kinase inhibitor, is a different substance able to increase ROS amounts by altering mitochondrial respiration, and in a diverse set of leukemia patients, this molecule could overwhelm the onset of chemoresistance [71,72]. In fact, an increased ROS concentration not only causes programmed cell death, but also induces noncaspase-dependent necroptosis, which is able to disable chemoresistance due to apoptotic escape [73,74].

Comparable results have also been obtained by employing molecules of natural origin. Tingenone (TG) and 22-hydroxytingenone (22-HTG) are compounds extracted from the plant *Salacia impressifolia* that are capable of inducing cell death in numerous types of neoplastic cells. In one study, the anti-AML effect of TG and 22-HTG was evaluated in the AML HL-60 cell line. Both substances displayed elevated toxicity against leukemic cells [75]. TG and 22-HTG decreased cell proliferation and induced the loss of mitochondrial potential and the destruction of internucleosomal DNA in HL-60 cells. Furthermore, the incubation of cells with a caspase inhibitor avoided TG- and 22-HTG-induced programmed cell death, suggesting that the cell death was due to apoptosis through a caspase-dependent system. Furthermore, the study of numerous genes has revealed that the two molecules were able to induce the alteration of the cellular antioxidant system, including a decrease in thioredoxin levels. This hypothesis was confirmed by the result that the use of N-acetylcysteine (NAC), a well-known antioxidant, completely stopped cell death induced by TG and 22-HTG. In addition, TG and 22-HTG caused DNA double-strand breaks and epigenetic changes of JNK2 and p38α, while the use of JNK/SAPK inhibitors or p38 MAPK inhibitors in some measure avoided cell death [75].

Similar effects have been recognized for 20-hydroxycinnamaldehyde (20-HCA), which is a compound extracted from *Cinnamomum cassia* [76]. 20-HCA has anticancer effects, including the inhibition of cell growth and the stimulation of programmed cell death [77,78]. As for the mechanism, 20-HCA caused cell death in HL-60 cells via the stimulation of different systems including the transfer of Bim and Bax from the cytosol to the mitochondria, reduction in Bcl-2 protein production, reduction of mitochondrial membrane potential, caspase stimulation, and stimulation of different kinases such as JNK and ERK1/2 [79]. Remarkably, 20-HCA induced an increase in ROS and reduction of GSH and protein thiols in leukemic cells. The use of antioxidants such as NAC blocked 20-HCA-induced transfer of Bim to the mitochondria and JNK phosphorylation, confirming that OS is necessary for 20-HCA-induced leukemic cell death. In vivo, animal experimental models showed that the intraperitoneal dispensation of 20-HCA reduced leukemic proliferation by increasing the production of nitrotyrosine and pro-apoptotic signals and decreasing the production of proliferating cell nuclear antigen protein [79].

Taken together, these results indicate that the pro-oxidant approach could be evaluated as a possible therapeutic strategy for acute myeloid leukemia.

### 2.2. Antioxidant Approach to Treat Leukemic Disease

Using antioxidants to combat the harmful consequences of ROS or toxicity caused by chemotherapy in several neoplastic conditions, including AML, was previously attempted in clinical practice. However, several studies stated that antioxidants may decrease the efficacy of antineoplastic drugs by safeguarding not only normal cells but also clonal cells [80].

Nevertheless, some experimentations demonstrated that when antioxidants are dispensed contemporarily with chemo-treatment, they may increase the toxicity of antineoplastic drugs to tumor cells and protect normal cells against therapy correlated injuries. Thus, antioxidant use may enhance the response rate to treatment and patient survival, without interfering with treatment [81,82]. Of course, the problem is establishing which dosage to employ, with the need to select a therapeutic dosage that blocks the proliferation of leukemic cells but does not influence normal cells.

NAC, often used as an antioxidant in vitro, was reported to increase the toxicity of different compounds via ROS-correlated and ROS-uncorrelated effects. Its mechanism of action is essentially ascribed to its capability to reduce disulfide bonds and to operate as a predecessor of GSH generation [83,84]. Nevertheless, this justification must be assumed with caution. NAC is an effective defense against hydroxyl radicals or hypochlorous acid, but it does not function with superoxide, the most important ROS, and it only interacts moderately with H_2_O_2_ [85,86,87].

In a study, NAC itself was reported to stimulate massive ROS generation in U937 and HL-60 leukemia cells [88]. In HL-60 cells, NAC stimulated NOX2 to generate superoxide, and its successive transformation into H_2_O_2_ by superoxide dismutase (SOD) 1, and SOD3 and the generation of ClO^−^ from H_2_O_2_ was essential for the onset of cell death. Adding extracellular SOD augmented NAC-induced cell death, while the addition of extracellular catalase blocked cell death. In U937 cells, the low toxicity induced by NAC was possibly induced by low-level production of SOD and NOX2. Nevertheless, also in this case, the addition of extracellular SOD caused cell death, and this result might be counterbalanced by extracellular catalase.

Thus, the fate of the cells is determined by the production of enzymes that regulate the generation and scavenger of ROS, and the modality of cell death after NAC administration assumes apoptotic and apoptotic-like characteristics in both cell lines [88].

Nevertheless, other compounds with antioxidant capabilities have been successfully used in the treatment of AML. Kushen or *Sophora flavescens Aiton* is a traditional Chinese medicine that has been evaluated as a possible the therapy for AML. Kushen displayed the ability to modifying ROS concentrations to avoid AML relapse with an antioxidant effect. An experiment demonstrated that Kushen administration reduced the growth of AML cells and the stimulated programmed cell death of AML cells. Furthermore, Kushen reduced intracellular ROS amounts by increasing peroxiredoxin 2 and peroxiredoxin 3 synthesis and reducing thioredoxin 1 generation. Laser confocal microscopy revealed that the proteins peroxiredoxin-2 could be networked with thioredoxin 1 by compound Kushen injection. In vivo, survival was longer, and the disease was moderately alleviated by reduced CD45+ immunophenotyping in peripheral blood in the compound Kushen injection-treated group in this AML model [89].

From previous research, it has emerged that the role of antioxidants in the treatment of leukemic disease still needs to be evaluated with extreme caution and that further studies are necessary to fully understand any beneficial effects or risks inherent in such use. It is likely that the effects of the administration of antioxidants are strictly dependent on the experimental conditions, the dosage used, and the cell type involved. Therefore, it appears necessary to be extremely cautious in the use of these substances in leukemia and an accurate risk–benefit assessment.

## 3. Oxidative Stress and Acute Lymphoblastic Leukemia

Acute lymphoblastic leukemia (ALL) is a hematological malignancy deriving from B- or T-lymphoid progenitor cells. It is the most frequent childhood tumor, accounting for about 25% of childhood tumors while a second peak in incidence happens after 50 years of age. ALL should be regarded as a group of heterogeneous conditions that need tailored treatment. Several factors may modify the biology of the disease. Host elements comprise gender, age, and ethnicity. Other factors are related to disease characteristics, including white blood cell count at diagnosis, immunophenotype, genetic profile, and extramedullary involvement. It should be noted that risk elements are not absolute, and they vary in meaning according to the treatment regimen [90]. A further factor capable of modifying the biological characteristics of the disease is constituted by the variations in the redox system.

As in the case of AML, ALL also appears to be characterized by the presence of abnormal intracellular oxidative stress. The most common genetic alteration in ALL patients is represented by the breakpoint cluster region-Abelson (BCR/ABL), with an incidence of 20–40% [91]. This fusion gene produces transcript with tyrosine kinase activity, stimulates cell growth, and reduces programmed cell death [92]. Numerous papers have reported that cells expression BCR-ABL oncoprotein have an accompanying increase in intracellular ROS [93]. Reports have demonstrated that BCR/ABL can increase ROS generation via the regulation of the NOX complex [94]. Moreover, BCR-ABL can also stimulate the PI3K/AKT/mTOR system to increase intracellular ROS synthesis [93]. With respect to normal cells, BCR/ABL-positive cells present more oxidative DNA injury, but display an enhanced capability to resist DNA damage [95].

In an animal experimental model of ALL, genetic analysis revealed the anomalous production of Janus kinase 3 (JAK3) and Aiolos (a member of Ikaros zing-finger family transcription factors generated by *Ikzf3*), which has been reported to be strictly correlated to the onset of ALL [96,97]. Lim et al. stated that several mutations were correlated with ROS-induced DNA damage. In an in vivo animal experimental model, secondary drivers of leukemogenesis in the Mb1- CreΔPB mouse model were evaluated using whole exome sequencing. The authors found that 5/8 leukemias had alterations in Jak3, 2/8 had mutations in Jak1, and 3/8 had mutations in Ikzf3. Genetic alterations with the highest variant allele frequency (VAF) presented C to T transition mutations that were matched with activation-induced cytidine deaminase, while the greater part of genetic alterations, with the smallest VAF, were characterized by C to A transversions correlated with ROS. Leukemic cells were reliant on high concentrations of ROS, directed by IL-7-dependent JAK-STAT signaling and modified antioxidant gene expression, which induced an 8-oxoguanine DNA injury. Adding the JAK inhibitor ruxolitinib can postpone leukemia development, decrease ROS and ROS-induced gene expression, suggesting that JAK mutations can modify the history of clonal evolution via ROS-induced DNA injury [98].

In addition, the biochemical parameters of ALL subjects demonstrated a relevant increase in the concentrations of malondialdehyde (MDA), a well-known indicator for OS [99], and an important increase in 8-oxo-7,8-dihydro-2′-deoxyguanosine and 8-hydroxy-2′-deoxyguanosine, markers of oxidative DNA injury in urine [100,101]. Finally, the amount of oxidatively changed DNA bases in lymphocytes from ALL patients was significantly greater than that in normal subjects [102,103,104].

The occurrence of OS in ALL patients could also be relevant from a prognostic point of view. Clonal diseases with MLL alterations, such as ALL, are correlated with poor prognosis. The genesis of MLL-fusion clonal diseases has been connected to the increased expression of *HOX/MEIS1* genes. A study used *Meis1*-knockout animals combined with *MLL-AF9* knock-in mice. The results displayed that *Meis1* is relevant for progress of established leukemia. Furthermore, authors demonstrated that *Meis1* loss induced augmented OS, oxygen flux, and programmed cell death [105], and that *hepatic leukemia factor (HLF*) was a target gene of *Meis1*. Hypoxia or *HLF* increased the OS, rescuing leukemia progression in *Meis1*-lacking cells. Therefore, the leukemia-driving abilities of *Meis1* are at least partly due to a low OS, supported by HLF.

These findings suggest that stimulating oxidative metabolism could have a therapeutic capacity in leukemia therapy. However, only few clinical trials have evaluated the impact of oxidative stress and its modulation on the efficacy of therapeutic treatment or on the onset of side effects of chemotherapy (Table 1).

### Modulation of Oxidative Stress to Treat ALL Leukemia

Altering the redox equilibrium of ALL cells increases the ROS concentration to stimulate programmed cell death, which is an efficient system to destroy leukemic cells. Drugs generally employed for the therapy of ALL include mitotic blockers such as vincristine and anthracyclines such as doxorubicin and daunorubicin. These compounds can perform their therapeutic action by stimulating ROS generation. However, several experiments show that a multiplicity of different substances could alter the oxidative regulation to increase the destruction of clonal cells, including histone deacetylase inhibitors (HDACIs), proteasome inhibitors, heme oxygenase-1 inhibitors such as protoporphyrin, and thioredoxin inhibitors [106]. Furthermore, thiopurines, which are the most relevant drugs employed in the maintenance therapy for ALL, may increase OS and this might be the system implicated in thiopurine-driven cytotoxicity [107,108].

Remarkably, ROS not only has a relevant effect on the stimulation of programmed cell death, but is also a powerful stimulant of autophagy [109,110,111]. Compounds with pro-oxidant effects, such as vincristine and vorinostat, lead to autophagy, increasing the ROS concentration in ALL cells; inhibiting autophagy significantly increases the cytotoxicity of chemotherapy, which corroborates that autophagy is an essential protective system for ALL cells during pro-oxidant therapy [112,113], and confirms the difficulty of modifying oxidative stress for therapeutic purposes.

Other drugs not generally used as chemotherapy might have a relevant effect on leukemic cells via their action on the redox balance. The contemporary administration of metformin (an oral hypoglycemic agent) and rotenone (a toxic substance that is the main chemical component of some plants) remarkably increased the chemosensitivity of Jurkat cells (an ALL cell line) via a mechanism implicating the production of hydrogen peroxide and anion superoxide radical [114]. Similarly, tigecycline, an antibiotic, is an interesting possibility for ALL treatment. It was reported that in ALL cells, tigecycline stimulates OS and the onset of an energy emergency by blocking mitochondrial respiration [115]. Fu et al. have demonstrated that tigecycline has a cytotoxic action on ALL cells, whereas its effects on normal HSCs are quite insignificant [115]. Other experiments state that the increased mitochondrial genesis and enhanced oxygen utilization in ALL cells with respect to normal HSCs might be the cause for the differing responses of the two types of cells to the antibiotic [116].

A therapeutic result can be achieved through a different approach acting on the variation in antioxidant systems. Schoeneberger et al. demonstrated that reducing GSH with buthionine sulfoximine (BSO) to decrease the antioxidative protection of ALL cells can increase ROS generation and stimulate the programmed cell death of ALL cells. This effect is obtained with the combined administration of a Smac mimetic, BV6 [116]. In contrast, this treatment did not work on normal cells, indicating that BSO has tumor selectivity. Other studies demonstrated that SOD blockers such as ATN-224 and 2-methoxyestradiol [117,118] present antileukemic properties and that 2-methoxyestradiol destroys ALL cells without altering normal hematopoietic stem cells. Similarly, auranofin, a blocker of antioxidant enzymes, also has high specificity for ALL cells [119].

Furthermore, old drugs have been modified to enhance their effectiveness via an increased effect of OS. For instance, nitrogen mustard is one of the oldest substances employed to treat hematological malignancies and has an antileukemic effect by increasing ROS concentrations [120,121]. Chen et al. covalently bound nitrogen mustard and boronic acid to create a ROS-stimulated nitrogen mustard and showed that this substance had remarkably increased cytotoxicity to leukemia cells [122]. Analogously, Liao et al. produced an HDACI prodrug by employing aryl boronic acid, which can be activated by increased ROS concentrations to release HDACIs, thus obtaining extremely effective therapeutical results [123]. In the same line of research, a study proposed the aryl boronic ester aminoferrocene prodrug, which can stimulate ROS production and block the antioxidant mechanism of leukemic cells, and reported no toxicity in healthy cells [124,125,126,127].

Finally, ALL includes a diverse group of hematologic diseases, resulting from different genetic aberrations in the initial lymphocyte expansion; in addition to the more usual form of B-ALL, there is the rarer form T cell-derived ALL (T-ALL). Acute T-ALL represents about 25% of adult ALLs. Even if the treatment of T-ALL has progressed in the last years, the elevated mortality of T-ALL patients necessitates the development of novel and efficacious treatments [128].

In the case of T-ALL, the modulation of oxidative stress and ROS production is also a promising therapeutic approach. For instance, poricoic acid A (PAA) is a component of the mushroom *Poria cocos*, and exercises regulatory actions on oxidative stress and inflammation via several signaling pathways [129].

A study demonstrated that PAA intensely decreased the cell vitality of T-ALL cells, and induced programmed cell death in vitro. Remarkably, PAA-reduced cell vitality and -triggered programmed cell death were ROS-dependent. Furthermore, PAA administration also induced ferroptosis in T-ALL cells with decreased GSH concentrations and augmented MDA levels [130].

The relationship between ROS and multiple signaling pathways is also interesting. mTOR stimulation is a common finding of T-ALL. Silic-Benussi et al. evaluated the correlation between the mTOR pathway and redox equilibrium employing inhibitors and gene silencing [131]. In vitro experiments performed on T-ALL cells and steroid-resistant patient-originated T-ALL xenograft cells showed that the mTOR inhibitor everolimus augmented ROS concentrations, increased lipid peroxidation, and stimulated the ROS-regulated transcription factor NRF2. These actions were associated with a reduction in the concentrations of NADPH and of glucose-6-phosphate dehydrogenase, which is the principal source of cytosolic NADPH required for conserving the cellular ROS-scavenging ability. Interestingly, everolimus caused mitochondrial membrane depolarization and dosage-dependent programmed cell death of T-ALL cells, but did not destroy normal T-cells. Furthermore, the combined use of everolimus and dexamethasone had a synergistic action on destroying T-ALL cells, while the results of mTOR blockade were diminished by ROS scavengers.

As reported above, mitochondria have been extensively evaluated as an objective for antileukemic therapy as they are implicated in ROS production, and a novel group of small membrane-permeable antileukemic substances, called “mitocans,” has been proposed. Mitocans operate at diverse levels comprising ROS generation and scavenging and have been employed in the studies on T-ALL treatment [132].

Moreover, the specificity and efficiency of mitocans may be augmented by chemical variation, supporting their intramitochondrial deposit. A usual modality is their link to a membrane-permeable cation, such as triphenylphosphonium, which tend to locate mitocans at the interface between mitochondrial matrix and inner mitochondrial membrane, thereby augmenting the overall antileukemic effectiveness. For example, the mitochondrial-targeted mitoVES is up to fifty times more effective in inducing programmed cell death in Jurkat cells with respect to the original compound [133].

## 4. Oxidative Stress and Onset of Side Effects from Chemotherapy in AML and ALL Patients

If the increase in OS induced by antileukemic treatment is certainly useful to destroy leukemic cells, this enhancement is also accountable for the onset of collateral effects of the treatment. In fact, an increased ROS concentration not only destroys the clonal cells, but also inopportunely induces damage to and death of normal HSCs, and Tang et al. demonstrated that the increased ROS concentration caused by the treatment also altered the BM niche where HSCs are located, damaging the hematopoietic capacity of BM [134]. The long-lasting action of OS on DNA can also induce aging and reduction of the self-renewal capability of HSCs. This could be relevant for the onset of BM failure [135].

Particularly interesting from this point of view could be the action of certain reactive species, such as chlorine species, including hypochlorous acid (HOCl), which can cause damage by reacting with biological molecules. These effects could be relevant to the induction of vascular and neurologic damage [136]. In fact, several reports displayed that chemotherapy treatment can cause alteration of the oxidative stress not only in the peripheral or medullary blood, but also in other areas.

A study selected a set of clinical signs (pain, depression, nausea, fatigue, sleep alteration) recognized to arise after treatment in leukemic patients according to the Childhood Cancer Symptom Cluster–Leukemia (CCSC-L) and stated that the onset of these symptoms causes obvious deterioration in the quality of life [137,138]. Moreover, many young patients suffer alteration in cognitive function. A study evaluated the relationships between markers of OS in the cerebrospinal fluid, the CCSC-L, and cognition in ALL patients before and after chemotherapy [139]. The results demonstrated that, when GSH markers were elevated (less OS) the CCSC-L severity was lower, and cognitive function tended to improve over time [139]. This fact is certainly interesting, as assessing ALL patients for elevated OS before stating chemotherapy would allow identification patients with elevated risk for cognitive impairments.

In an analogous study, antioxidants were evaluated from cerebrospinal fluid longitudinally during the treatment of leukemia patients [140]. Results highlighted that GSH/GSSG ratios were linked to fatigue scores during the therapy as patients with low ratios in the first time of treatment presented greater fatigue scores during induction and post-induction treatment [140]. These results suggest that increased OS during more intensive periods of chemotherapy may justify the occurrence of patients reporting fatigue.

Jonas et al. confirmed that leukemic subjects getting high-dosage treatment presented a 20% reduction in plasma GSH at the beginning and during 2 weeks after treatment [141]. Two different trials demonstrated that leukemic subjects who were treated with chemotherapy and GSH showed an improved quality of life, reduced therapy-correlated side effects, and reduced neurotoxicity with respect to patients who were treated with chemotherapy and placebo [142,143].

A further analysis studied the fatigue suffered by leukemic patients during the first period of chemotherapy [144]. An established biomarker of nitrosative stress, protein 3-nitrotyrosine (3NT) residues, was longitudinally assessed in the cerebral spinal fluid, and more severe symptoms were correlated with greater 3NT concentrations at the start of the treatment [144].

These results were authenticated by other studies, and essential variances were seen in the number of symptoms presented longitudinally during the various periods of treatment in ALL subjects, with the effect of OS on symptom occurrence recognized [145].

A particularly interesting study has assessed the relationship between OS and diet in young leukemic patients during therapy [146]. The evaluation of nutritional intake and OS was performed before and during chemotherapy. OS increased in all leukemic subjects, according to chemotherapy-induced oxidative effects. Dietary analysis demonstrated a correlation with the intake of protein (animal, vegetable, and total protein) [146]. These results indicate the possibility that nutritional protein could affect clinical outcomes.

However, the relationship between dietary protein source, pro-oxidant production, antioxidant production, and leukemia outcome is still uncertain, and it is possible that vegetable and dairy proteins may have diverse effects on ROS production. In depth analysis into the effect of specific amino acids or particular forms of protein sources is needed to improve the effects of chemotherapy and to enhance outcomes for leukemia subjects via dietary adjustment.

As stated above, the brain is intensely susceptible to the negative side effects of chemotherapy treatment [147] as it is extremely vulnerable to OS due to inadequate antioxidant ability and greater energy demand [148]. Furthermore, the brain contains a high quantity of lipids [149], and the OS-induced generation of lipid peroxides may cause deep damage to membrane functions [150]. Chemotherapy increases the central nervous system OS as evaluated by cerebrospinal fluid (CSF) 8-hydroxy-2′-deoxyguanosine (8-OH-dG) concentrations, with the amount being proportionate to the severity of therapy. Patients with brain toxicity presented only a slight increase of CSF 8-OH-dG levels with respect to patients without toxicity [151].

Thus, the evaluation of redox elements could inform the recognition of subjects at greater risk of onset for collateral effects and the treatment of antioxidants to decrease the damages of chemotherapy treatment.

## 5. Future Perspectives

The possibility of greater and more refined modulation of oxidative stress could achieve ever more selective targeting of neoplastic cells, allowing the induction of more effective and less toxic responses for the patient. The use of adequate modulators could allow a reduction in the dosage of traditional chemotherapies, a reduction in chemoresistance, and better control of the minimal residual disease in these patients.

However, from the above, the homogeneity of behavior of the leukemic disease could emerge with regard to the alterations of oxidative stress and the cellular response to these changes. In contrast, specific responses of different leukemic cells to the modulation of oxidative stress has been demonstrated, with studies showing significant differences between ALL-B, ALL-T, and AML cells.

In fact, the genesis of different forms of leukemia implicates the aberrant stimulation of different signaling pathways able to regulate the growth, differentiation, and survival of leukemic cells; depending on the cell sub-type, the abnormal ROS production and the changes in antioxidant levels may be different in diverse leukemia cells.

The specific state of the oxidative and antioxidation systems can in fact be determined by genetic and metabolic conditions peculiar to each leukemic form. For instance, it was reported that FLT3-ITD, a genetic mutation specific to certain types of AML, stimulates ROS production [42], while other studies demonstrated that the administration of stress inducers stimulated augmented NRF2 activation in AML cell lines but not in other leukemic cells [152].

In a study, leukemic patients were divided according to the type of leukemia and displayed extreme variance among themselves and compared with normal subjects [99]. For instance, ALL-T patients had augmented concentrations of MDA with respect to other form of leukemia, such as ALL-B and AML. Reduced amounts of antioxidant enzymes are present in all leukemic patients, but SOD concentrations were different based on disease type, as ALL-T patients had diverse levels of SOD with respect to AML patients and ALL-B subjects. Furthermore, ALL-T patients presented an increased concentration of GSH compared with AML and ALL-B patients. Similarly, ALL-T patients had increased catalase and NO with respect to other sub-types [99].

All this could easily explain the different responses of the different leukemic cells to the administration of substances capable of modifying oxidative stress.

For instance, myricitrin, vitexin, and vanillin were extracted and have been recognized as the functional components responsible for the anti-growth effect of *M. longifolia*, *P. cineraria*, and *F. indica*. These substances displayed dosage-dependent toxicity against leukemic cells through ROS and NO generation, SOD depletion, and augmented lipid peroxidation. Although the effects of these compounds were analogous in diverse forms of leukemia cells, the substances had different efficacy when applied to AML, CML, and ALL cells [153].

It is therefore evident that only complete of the biology of the various diseases and of the state of the redox system in the different forms of leukemia will allow the modulation of oxidative stress to be fully utilized as a method of treating leukemic disease. The comprehension of the subtle differences related to the different oxidative states and the different response of leukemic cells to ROS may allow optimization of the results obtained from the manipulation of the oxidative stress in leukemic patients.

## 6. Conclusions

The accrued findings suggest that modulation of the redox balance is central characteristic of leukemia. The change in this balance in leukemia cells is commonly due to the presence of oxidative stress, indicating that leukemia cells may be more susceptible to variations in ROS concentrations compared with healthy cells. Still, the conventional chemotherapy approaches for the treatment of leukemia still have numerous shortcomings. Thus, new treatment strategies employing oxidants or antioxidants should be investigated, and have been applied in pre-clinical and clinical practice.

Although unrestrained cell exposure to redox imbalance may induce cytotoxicity implicating normal cells, the specific stimulation of toxicity in leukemic cells through a coordinated pro-oxidant or antioxidant treatment could help to obtain an effective and secure treatment for acute leukemia. The increasing understanding of novel technologies and the deepening knowledge obtained through preclinical and clinical studies of innovative approaches will help to develop the safe design of redox-based leukemia therapy.

## Figures and Tables

**Table 1:** Clinical trials evaluating the impact of oxidative stress on therapeutic treatment or on the onset of side effects of chemotherapy. www.clinicaltrials.com (accessed on 15 May 2022).

NCT Number	Study Title	Condition	Study Type	Status
NCT04488237	Vitamin D and Methotrexate Adverse Effects	Acute lymphoblastic leukemia	Observational	Not yet recruiting
NCT02373579	Effect of Omega-3 Fatty Acids on Methotrexate Induced Hepatotoxicity in Children with Acute Lymphoblastic Leukemia	Acute lymphoblastic leukemia	Interventional	Completed
NCT00671697	Decitabine, Arsenic Trioxide and Ascorbic Acid for Myelodysplastic Syndromes and Acute Myeloid Leukemia	Myelodysplastic syndromes and acute myeloid leukemia	Interventional	Completed
NCT02361047	Let’s Play! Healthy Kids After Cancer	Childhood acute lymphoblastic leukemia in remission	Interventional	Completed
NCT03467386	Total Marrow and Lymphoid Irradiation Before Donor Transplant and Cyclophosphamide in Treating Patients with Acute Myeloid Leukemia	Acute myeloid leukemia	Interventional	Active not recruiting

## Data Availability

Data is contained within the article.
